# Supplementary material for: Interrogating basal ganglia circuit function in people with Parkinson’s disease and dystonia
Source: eLife. 2024 Aug 27;12:RP90454. doi: 10.7554/eLife.90454 (PMC11349293; doi:10.7554/eLife.90454)
Supplement: Supplementary file 1. — The table provides a summary of the patient data included in the study. It details the diseases studied, the types of data collected (e.g., neuronal, plasticity), and the corresponding clinical scores. [file elife-90454-supp1.docx]

**Supplementary File 1**

| **ID** | **disease** | **plasticity tasks** | **neurons** | **scale** | **total clinical score** | **hypokinetic clinical score** |
| --- | --- | --- | --- | --- | --- | --- |
| 1 | Parkinson’s disease | - | 5 | UPDRSIII | 34 | 14 |
| 2 | Parkinson’s disease | - | 3 | UPDRSIII | 32 | 10.5 |
| 3 | Parkinson’s disease | - | 7 | UPDRSIII | 48 | 17 |
| 4 | Parkinson’s disease | - | 6 | UPDRSIII | 39 | 16 |
| 5 | Parkinson’s disease | - | 1 | UPDRSIII | 40 | 19 |
| 6 | Parkinson’s disease | - | 7 | UPDRSIII | 47 | 23 |
| 7 | Parkinson’s disease | - | 5 | UPDRSIII | 45.5 | 17 |
| 8 | Parkinson’s disease | - | 10 | UPDRSIII | 31 | 14 |
| 9 | Parkinson’s disease | - | 12 | UPDRSIII | 34 | 11 |
| 10 | Parkinson’s disease | - | 9 | UPDRSIII | 29 | 11 |
| 11 | Parkinson’s disease | - | 7 | UPDRSIII | 26 | 10 |
| 12 | Parkinson’s disease | - | 3 | UPDRSIII | 46 | 21 |
| 13 | Parkinson’s disease | - | 4 | UPDRSIII | 37 | 18.5 |
| 14 | Parkinson’s disease | - | 2 | UPDRSIII | 48 | 17 |
| 15 | Parkinson’s disease | - | 5 | UPDRSIII | 29 | 8 |
| 16 | Parkinson’s disease | - | 2 | UPDRSIII | 68 | 27 |
| 17 | Parkinson’s disease | - | 3 | UPDRSIII | 31 | 11 |
| 18 | Parkinson’s disease | - | 4 | UPDRSIII | 17 | 5 |
| 19 | Parkinson’s disease | - | 1 | UPDRSIII | 31 | 12 |
| 20 | Parkinson’s disease | 1 | 3 | UPDRSIII | 23 | 12 |
| 21 | Parkinson’s disease | - | 6 | UPDRSIII | 35 | 15 |
| 22 | Parkinson’s disease | - | 8 | UPDRSIII | 32 | 10.5 |
| 23 | Parkinson’s disease | - | 1 | UPDRSIII | 13.5 | 3.5 |
| 24 | Parkinson’s disease | - | 5 | UPDRSIII | 43 | 15 |
| 25 | Parkinson’s disease | - | 3 | UPDRSIII | 31.5 | 10 |
| 26 | Parkinson’s disease | 2 | - | UPDRSIII | - | - |
| 27 | Parkinson’s disease | - | 3 | UPDRSIII | 65.5 | 32 |
| 28 | Parkinson’s disease | 1 | 2 | UPDRSIII | 38 | 16 |
| 29 | Parkinson’s disease | - | 1 | UPDRSIII | 32 | 13 |
| 30 | Parkinson’s disease | 3 | 8 | UPDRSIII | 40.5 | 19 |
| 31 | Parkinson’s disease | 1 | 9 | UPDRSIII | 24 | 11 |
| 32 | Parkinson’s disease | - | 14 | UPDRSIII | 23 | 7 |
| 33 | Parkinson’s disease | 1 | 6 | UPDRSIII | 60 | 22 |
| 34 | Parkinson’s disease | 1 | - | UPDRSIII | - | - |
| 35 | Parkinson’s disease | 1 | 3 | UPDRSIII | 30 | 7 |
| 36 | Parkinson’s disease | - | 4 | UPDRSIII | 29 | 11 |
| 37 | Parkinson’s disease | - | 3 | UPDRSIII | 46 | 19 |
| 38 | Parkinson’s disease | - | 6 | UPDRSIII | 26.5 | 9 |
| 39 | Parkinson’s disease | - | 9 | UPDRSIII | 37.5 | 18 |
| 40 | Parkinson’s disease | 2 | 3 | UPDRSIII | 49.5 | 23 |
| 41 | Parkinson’s disease | - | 3 | UPDRSIII | 57.5 | 24 |
| 42 | Parkinson’s disease | - | 4 | UPDRSIII | 59 | 20 |
| 43 | Parkinson’s disease | - | 4 | UPDRSIII | 35.5 | 12.5 |
| 44 | Parkinson’s disease | - | 4 | UPDRSIII | 68 | 26.5 |
| 45 | Parkinson’s disease | 1 | 8 | UPDRSIII | 41 | 16.5 |
| 46 | Parkinson’s disease | - | 6 | UPDRSIII | 32.5 | 15 |
| 47 | tardive dystonia | - | 2 | BFMDRS | 12 | n/a |
| 48 | cervical dystonia | 1 | 8 | TWSTRS | 22 | n/a |
| 49 | segmental dystonia | 2 | 7 | BFMDRS | 21 | n/a |
| 50 | hemidystonia | 1 | 0 | BFMDRS | 27 | n/a |
| 51 | generalized dystonia | - | 2 | BFMDRS | 11 | n/a |
| 52 | generalized dystonia | 1 | 0 | BFMDRS | 35 | n/a |
| 53 | generalized dystonia | 1 | 0 | BFMDRS | 27 | n/a |
| 54 | generalized dystonia | - | 4 | BFMDRS | 36 | n/a |
| 55 | craniocervical dystonia | - | 7 | TWSTRS | 22 | n/a |
| 56 | cranial dystonia | 3 | - | BFMDRS | 32 | n/a |
| 57 | cervical dystonia | - | 8 | TWSTRS | 29 | n/a |
| 58 | cervical dystonia | - | 5 | TWSTRS | 19 | n/a |
| 59 | cervical dystonia | - | 9 | TWSTRS | 3 | n/a |
| 60 | cervical dystonia | - | 18 | TWSTRS | 15 | n/a |
| 61 | cervical dystonia | - | 6 | TWSTRS | 4 | n/a |
| 62 | cervical dystonia | 1 | 10 | TWSTRS | 34 | n/a |
| 63 | cervical dystonia | - | 12 | TWSTRS | 13 | n/a |
| 64 | cervical dystonia | - | 10 | TWSTRS | 20 | n/a |
| 65 | cervical dystonia | - | 5 | TWSTRS | 35 | n/a |
| 66 | cervical dystonia | 2 | 6 | TWSTRS | 14 | n/a |
| 67 | cervical dystonia | - | 6 | TWSTRS | 8 | n/a |
| 68 | cervical dystonia | - | 8 | TWSTRS | 16 | n/a |
| 69 | axial dystonia | - | 3 | BFMDRS | 3 | n/a |
