# Supplementary material for: Interrogating basal ganglia circuit function in people with Parkinson’s disease and dystonia
Source: eLife. 2024 Aug 27;12:RP90454. doi: 10.7554/eLife.90454 (PMC11349293; doi:10.7554/eLife.90454)
Supplement: Supplementary file 2. — The table compares neuronal features such as firing rates, patterns, and oscillations between PD and dystonia patients. [file elife-90454-supp2.docx]

**Supplementary File 2**

| **feature** | **dystonia** (based on 135 neurons from 19 patients) | **PD** (based on 222 neurons from 44 patients) |
| --- | --- | --- |
| firing rate (Hz) | 70.57 ± 19.39 | 83.08 ± 16.46 |
| burst index | 5.40 ± 1.42 | 4.21 ± 1.27 |
| coefficient of variation | 0.54 ± 0.09 | 0.44 ± 0.07 |
| theta (dB) | -10.88 ± 0.93 | -10.86 ± 1.68 |
| alpha (dB) | -12.48 ± 1.10 | -12.70 ± 1.27 |
| low beta (dB) | -12.85 ± 1.04 | -13.29 ± 0.95 |
| high beta (dB) | -12.51 ± 0.51 | -12.74 ± 1.33 |
