# Supplementary material for: Interrogating basal ganglia circuit function in people with Parkinson’s disease and dystonia
Source: eLife. 2024 Aug 27;12:RP90454. doi: 10.7554/eLife.90454 (PMC11349293; doi:10.7554/eLife.90454)
Supplement: Supplementary file 3. — (a) The table provides Bonferroni-corrected statistics for comparing neuronal features across different diseases, and (b) reports the Benjamini–Hochberg false discovery rate-corrected statistics for correlating neuronal features with clinical scores. [file elife-90454-supp3.docx]

**Supplementary File 3a**

| **test** | **independent variable** | **dependent variable (neuronal feature)** | **original p-value** | **corrected p-value** |
| --- | --- | --- | --- | --- |
| 1 | disease | firing rate | 0.0252 | 0.1746 |
| 2 | disease | burst index | 0.0024 | 0.0168 |
| 3 | disease | coefficient of variation | 7.46e-5 | 0.0005 |
| 4 | disease | theta power | 0.6265 | 1 |
| 5 | disease | alpha power | 0.3649 | 1 |
| 6 | disease | low beta power | 0.1176 | 0.8232 |
| 7 | disease | high beta power | 0.4585 | 1 |

**Supplementary File 3b**

| **test** | **independent variable (neuronal feature)** | **dependent variable (clinical score)** | **original p-value** | **corrected p-value** |
| --- | --- | --- | --- | --- |
| 1 | theta power | dystonia | 0.0242 | 0.17892 |
| 2 | low beta power | PD hypokinetic | 0.033 | 0.17892 |
| 3 | coefficient of variation | dystonia | 0.0364 | 0.17892 |
| 4 | firing rate | dystonia | 0.037 | 0.17892 |
| 5 | low beta power | PD total | 0.0426 | 0.17892 |
| 6 | high beta power | PD hypokinetic | 0.0698 | 0.225 |
| 7 | alpha power | PD total | 0.075 | 0.225 |
| 8 | alpha power | PD hypokinetic | 0.0902 | 0.236775 |
| 9 | high beta power | PD total | 0.111 | 0.259 |
| 10 | burst index | dystonia | 0.1748 | 0.36708 |
| 11 | alpha power | dystonia | 0.3898 | 0.744163636 |
| 12 | theta power | PD total | 0.5666 | 0.99155 |
| 13 | firing rate | PD total | 0.6666 | 1 |
| 14 | coefficient of variation | PD total | 0.6828 | 1 |
| 15 | coefficient of variation | PD hypokinetic | 0.757 | 1 |
| 16 | firing rate | PD hypokinetic | 0.832 | 1 |
| 17 | burst index | PD total | 0.903 | 1 |
| 18 | low beta power | dystonia | 0.9054 | 1 |
| 19 | burst index | PD hypokinetic | 0.9402 | 1 |
| 20 | theta power | PD hypokinetic | 0.9924 | 1 |
| 21 | high beta power | dystonia | 1 | 1 |
